# Prevalence and appropriateness of indwelling urinary catheters in Japanese hospital wards: a multicenter point prevalence study

**DOI:** 10.1186/s12879-022-07162-3

**Published:** 2022-02-21

**Authors:** Kohta Katayama, Jennifer Meddings, Sanjay Saint, Karen E. Fowler, David Ratz, Yasuaki Tagashira, Yumi Kawamura, Tatsuya Fujikawa, Sho Nishiguchi, Naomi Kayauchi, Nobumasa Takagaki, Yasuharu Tokuda, Akira Kuriyama

**Affiliations:** 1 Department of General Medicine, International University of Health and Welfare Narita Hospital, 852 Hatakeda, Narita, Chiba 286-8520 Japan; 2 Center for Clinical Management Research, U.S. Department of Veterans Affairs Ann Arbor Healthcare System, Ann Arbor, MI USA; 3 Department of Internal Medicine, University of Michigan Medical School, Ann Arbor, MI USA; 4 Division of General Pediatrics, Department of Pediatrics and Communicable Diseases, University of Michigan, Ann Arbor, MI USA; 5 Division of Infectious Diseases, Tokyo Metropolitan Tama Medical Center, Tokyo, Japan; 6 Department of General Internal Medicine and Emergency Medicine, Mimihara General Hospital, Sakai, Osaka Japan; 7 Department of General Internal Medicine, Mitoyo General Hospital, Kanonji, Kagawa Japan; 8 Department of General Internal Medicine, Shonan Kamakura General Hospital, Kamakura, Japan; 9 Department of Infection Control, Mito Kyodo General Hospital, Mito, Ibaraki Japan; 10 Nobumasa Clinic, Kyoto, Japan; 11 Muribushi Okinawa Center for Teaching Hospitals, Urasoe, Okinawa Japan; 12 Emergency and Critical Care Center, Kurashiki Central Hospital, Kurashiki, Okayama Japan

**Keywords:** Urinary catheters, Infection control, Prevalence, Patient safety, Japan

## Abstract

**Background:**

Indwelling urinary catheters are commonly used in hospitalized patients, which can lead to the development of urinary catheter complications, including catheter-associated urinary tract infection (CAUTI). Limited reports on the appropriateness of urinary catheter use exist in Japan. This study investigated the prevalence and appropriateness of indwelling urinary catheters, and the incidence of CAUTI in non-intensive care unit (non-ICU) wards in Japanese hospitals.

**Methods:**

This prospective observational study was conducted in 7 non-ICU wards from 6 hospitals in Japan from October 2017 to June 2018. At each hospital the study teams evaluated urinary catheter prevalence through in-person bedside evaluation for at least 5 days of each week for 3 months. Catheter associated urinary tract infection (CAUTI) incidence and appropriateness of catheter use was collected via chart review.

**Results:**

We assessed 710 catheter-days over 5528 patient-days. The mean prevalence of indwelling urinary catheter use in participating wards was 13% (range: 5% to 19%), while the mean incidence of CAUTI was 9.86 per 1000 catheter-days (range: 0 to 33.90). Approximately 66% of the urinary catheter days assessed had an appropriate indication for use (range: 17% to 81%). A physician's order for catheter placement was present in only 10% of catheterized patients.

**Conclusion:**

This multicenter study provides epidemiological information about the appropriate use of urinary catheters in Japanese non-ICU wards. A multimodal intervention may help improve the appropriate use of urinary catheters.

**Supplementary Information:**

The online version contains supplementary material available at 10.1186/s12879-022-07162-3.

## Introduction

Indwelling urinary catheters are commonly used in hospitalized adults, including approximately 20% of hospitalized patients in Western countries [1, 2]. Urinary catheter complications are also common, including catheter-associated urinary tract infection (CAUTI) with a reported prevalence of 6% of hospital-associated infections (HAIs) in the United States [3] as well as causing patient discomfort from urethral trauma, immobility, and inadvertent removal [4]. The most effective intervention to prevent both infectious and non-infectious harms is avoiding inappropriate indwelling urinary catheter use [5, 6].

Limited reports on the appropriateness of urinary catheter use exist in Japan [7, 8]. One study from Japanese intensive care units (ICUs) reported that the point prevalence of urinary catheters was 76%, with only 54% of those catheters considered appropriate [7]. Another study conducted in Japanese stroke units reported inappropriate urinary catheter use was 50.1% [8]. However, appropriate urinary catheter use in other types of hospital units in Japan has not been previously studied. Given the infectious and non-infectious harms of indwelling urinary catheters, we wanted to estimate the prevalence and appropriateness of indwelling urinary catheters in non-critical care units in Japanese hospitals, and to determine the incidence of CAUTI in these units. Such a baseline study is necessary before undertaking a quality improvement intervention to limit the use of unnecessary urinary catheters in Japan.

## Methods

**Fig. 1:**
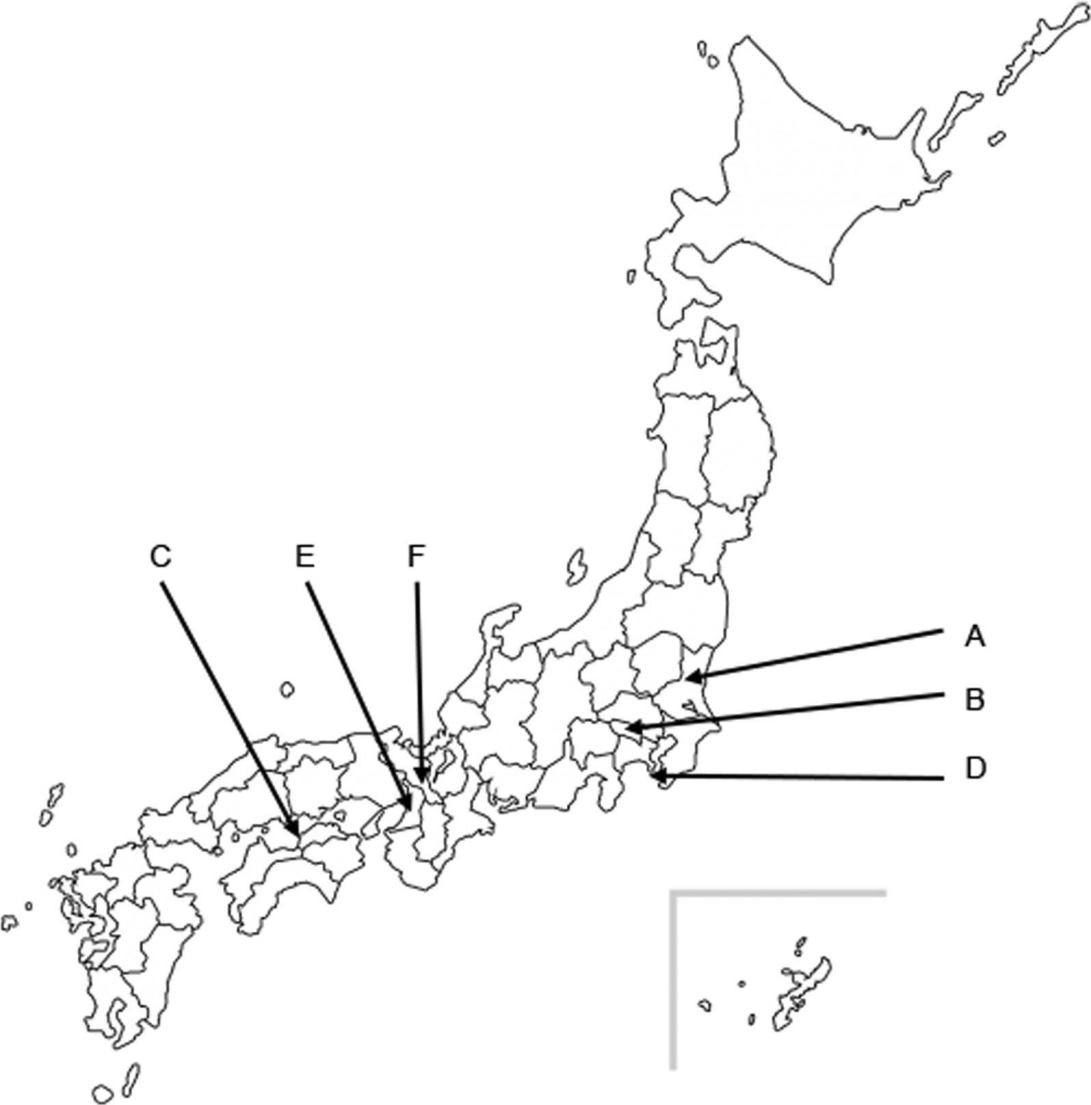
Participating hospital distribution

This prospective study was conducted at multiple hospitals in Japan from October 2017 to June 2018. In total, we enrolled 7 wards from 6 hospitals. All wards voluntarily participated in this study. The participating hospitals included one university-affiliated hospital (Hospital A), and a variety of public (Hospitals B & C) and private (Hospitals D, E, & F) hospitals. All participating hospitals were accredited by the Japan Council for Quality Health Care, with the accreditation approved under the International Accreditation Programme of the International Society for Quality in Health Care. Each participating unit had between 40 and 60 adult beds and received both emergency and scheduled admission. Additional participating hospital and unit characteristics can be found in Table 1. Hospital distribution is described in Fig. 1. Although all hospitals have an infection prevention and control (IPC) team, only Hospital B employed physicians specializing in infectious diseases. The ethics committee of each participating hospital approved the study protocol.

**Table 1:** Participating hospital characteristics

Hospital	Hospital type	Hospital Beds	Ward type	Ward Beds
Hospital A	University-affiliated	389	Respiratory medicine, neurology, otolaryngology	46
Hospital B	Public	789	Gastroenterology, gastroenterological surgery	44
Hospital C—Unit 1	Public	482	Internal medicine	41
Hospital C—Unit 2	Public	482	Internal medicine	44
Hospital D	Private	658	Internal medicine, surgery, urology, orthopedics, gynecology	45
Hospital E	Private	386	Internal medicine, orthopedics	47
Hospital F	Private	60	Internal medicine, orthopedics	60

Study observers—either physicians or research nurses—at each participating unit evaluated every admitted patient for urinary catheter prevalence, appropriateness of catheter use, and CAUTI incidence for one week (5 weekdays) per month for 3 months. This data collection period of 3 work weeks over 3 months for this descriptive assessment was determined by feasibility.

The study data collection tool was similar to those used for other studies and can be found in Additional file 1: Appendix S1 [7, 9, 10]. To assess prevalence, the observer rounded at fixed times and visited each patient on the ward each assessment day to visually confirm the presence or absence of a urinary catheter. When a catheter was present, the study observers would ask the bedside nurse for their assessment of catheter indication. Observers in all hospitals, except for Hospital C, also independently assessed the indication for every urinary catheter through medical record review. The medical records were also reviewed for orders for urinary catheter placement as well as documentation of urinary catheter presence. These methods were standardized between study sites to promote consistent data collection.

The 2009 Healthcare Infection Control Practice Advisory Committee (HICPAC) guidelines [11] and the Ann Arbor Criteria for Appropriate Urinary Catheter Use [6] were used to determine catheter appropriateness. The Ann Arbor Criteria was developed with a panel of expert clinicians in the United States, using all available world literature available at the time and applying the RAND/UCLA Appropriateness Method. Given the clinical conditions and urinary catheter products in the US and Japan are very similar for adult patients, these criteria should be applicable to hospitals in both countries.

The list of indications assessed is provided in Table 2. Indwelling urinary catheters in critically ill patients were deemed appropriate when medical staff required hourly urine volume measurement. Even in non-ICU wards, appropriate urine output monitoring by an indwelling urinary catheter was sometimes felt to be required to manage patients with electrolyte abnormalities or decompensated heart failure. Urinary catheters in patients who required prolonged strict immobilization for therapeutic purposes, such as pelvic fracture and unstable thoracic or lumbar spine, were also considered appropriate.

**Table 2:** Appropriate and inappropriate indwelling urinary catheter indications

Appropriate indications for catheter use	Inappropriate indications for catheter use
Acute urinary retention or bladder outlet obstruction	Incontinence
Need accurate input and output monitoring in critically ill patient	Immobility
Perioperative use	Monitoring input and output in non-critically ill patient
Urologic surgery or surgery on contiguous structures of genitourinary tract	
Anticipated prolonged duration of surgery	
Anticipated to receive large-volume infusions or diuretics during surgery	Patient or family request
Need for intraoperative monitoring of urinary output	
To assist with healing of open sacral or perineal wounds in incontinent patients	Convenience
Patient requires prolonged immobilizations, such as pelvic fracture	Confusion
To improve comfort care for end-of-life care	No apparent reason

Diagnostic criteria from the National Healthcare Safety Network of the Center for Disease Control and Prevention [12] was used to identify cases of CAUTI. CAUTI cases met all of the following criteria: 1) urinary catheter was inserted for more than 3 consecutive days and either present for any portion of the day of the event or removed the day before the event; 2) patient had more than one of the following symptoms: fever > 100.4 degrees F, suprapubic tenderness, costovertebral angle pain or tenderness; and 3) positive urine culture with more than 10^5^ colony-forming units/mL with less than 2 species of microorganisms identified.

The same observer at each unit conducted the rounds and chart review for the entire observation period. Study observers communicated with each other frequently to address any issues and ensure consistency with data collection between study sites. The data from each unit was entered into Excel for analysis.

### Statistical analyses

The primary outcomes were: (1) proportion of patients in non-ICU wards with an indwelling urinary catheter; (2) proportion of patients with a urinary catheter that had an appropriate indication based on independent assessment; and (3) CAUTI incidence. We also assessed how often the urinary catheter’s use was documented in the medical record, and if there was a physician order for placement of the urinary catheter. Data analysis was conducted through SAS software, version 9.4 (Cary, North Carolina).

## Results

Data were assessed for 710 catheter-days over 5528 patient-days. The prevalence of urinary catheters was 13% (range: 5% to 19%). Table 3 lists urinary catheter utilization by hospital. One hospital (Hospital C) did not conduct an independent review of urinary catheter indication and therefore their data on catheter appropriateness were excluded. Based on the observers’ independent assessment, urinary catheters were deemed appropriate in 371 of the 586 catheter-days (63%; range: 17% to 81%). The total incidence of CAUTI was 9.86 per 1000 catheter-days.

**Table 3:** Baseline urinary catheter point prevalence in Japanese hospitals

Hospital	Patient days	Catheter days	Point prevalence (%)	Documented in record	Order for placement	Appropriate Indications per bedside Nurse Assessment	Appropriate Indications per Independent Observer Assessment	CAUTI (per 1000 catheter-days)
Hospital A	826	41	5	8 (20%)	1 (2%)	17 (41%)	7 (17%)	0 (0)
Hospital B	717	134	19	134 (100%)	0 (0%)	100 (75%)	78 (58%)	0 (0)
Hospital C—Unit 1	564	54	10	51 (94%)	0 (0%)	50 (93%)	N/A*	0 (0)
Hospital C—Unit 2	588	70	12	70 (100%)	0 (0%)	49 (70%)	N/A*	1 (14.29)
Hospital D	925	128	14	119 (93%)	8 (6%)	101 (79%)	89 (70%)	2 (15.63)
Hospital E	893	118	13	115 (97%)	35 (30%)	65 (55%)	63 (53%)	4 (33.90)
Hospital F	1015	165	16	152 (92%)	26 (16%)	136 (82%)	134 (81%)	0 (0)
Total	5528	710	13	649 (91%)	70 (10%)	518 (73%)	371 (63%)	7 (9.86)

*IQR* interquartile range

^*^Independent assessment of indication by observer (i.e., research team physician or nurse) was not collected in Hospital C

The most common indication for urinary catheter use was acute urinary retention or bladder outlet obstruction (as assessed by bedside nurses (38%) and observers (35%)), followed by need for accurate measurement of input and output in critically ill patients (19% bedside nurses, 18% observers). Common indications for urinary catheters that were deemed inappropriate included monitoring input and output in non-critically ill patients (15% bedside nurses, 12% observers) and no apparent reason for catheter use (2% nurses, 12% observers). The majority of patients with catheters were medical patients (71.8%), followed by surgery patients (27.0%) and neurology patients (1.1%). Urinary catheters were inserted most frequently in each unit (42.8%), followed by the emergency room (26.5%) and operating room (11.7%). Although urinary catheters were documented in the medical record in 91% of patients, a physician's order for catheter placement was present in only 10% of records for catheterized patients.

## Discussion

Our multicenter study found a urinary catheter prevalence of 13%, with 63% of assessed catheter-days meeting appropriate indications for use. CAUTI incidence in our study was 9.86 infections per 1000 catheter-days. The urinary catheter prevalence found in these Japanese hospitals was similar to reports of urinary catheter prevalence in other countries, such as the U.S. (18.7–20.1%) [1, 3], Canada (22.4%) [2], The Netherlands (18.3%–21.2%) [13, 14], Australia (20.7%) [15], and Korea (14.9%) [16]. However, our findings were much improved from another study conducted in Japan which reported a urinary catheter prevalence of 27.1% in non-ICU settings [17]. One possible explanation for this improvement is that all of our participating hospitals were accredited by The Japan Council for Quality Health Care, indicating their focus on providing high quality health care. Therefore, our sample may not be representative of all hospitals in Japan, where only 26% of hospitals have received this accreditation [18]. This focus on quality may help explain the lower urinary catheter prevalence found in this study. Our sample also had a lower acuity of illness and a small number of perioperative patients, which likely contributed to fewer urinary catheters used.

The proportion of appropriate urinary catheter use was variable in our sample, with the average appropriate use lower than reports from several studies. For example, In the United States, the proportion of appropriate use of urinary catheters was reported to be 70.9%–73.1% in a multicenter study conducted in Emergency Departments [19]. One university hospital in the Netherlands reported that 89.2% of urinary catheters were appropriate when inserted, however the percentage of catheters with an appropriate indication decreased as catheter duration increased [20]. The same phenomenon was reported in hospitals in Korea. [16].

The CAUTI incidence (CAUTI per 1000 catheter-days) was also higher than reports from the US (1.54–2.28) [1], Korea (1.6) [16], and The Netherlands (4.0 infections per 1000 catheter-days) [14]. Multimodal approaches to promote prompt urinary catheter removal when no longer appropriate have shown success in both Japan and the US [1, 9, 10]. These approaches frequently include nurse-initiated catheter removal when deemed no longer appropriate. However, this practice is much more widely used in the US (59.1%; S. Saint, MD, unpublished data, February 2019) than in Japan (21% to 34%) [21]. Use of urinary catheter reminders or stop-orders have also been demonstrated to be effective in multiple settings [5], but are used routinely in only approximately 20% of Japanese hospitals [21]. Similar to a prior study conducted in Japanese ICUs [7] our study also found a lack of written physician orders for catheter placement. Promoting use of these types of strategies in Japanese hospitals may help reduce inappropriate use of urinary catheters and the occurrence of CAUTI.

Our study has some important limitations. Participating wards in this study represent a small sample of Japanese hospitals that all obtained accreditation from The Japan Council for Quality Health Care, and therefore findings may not be generalizable to all hospitals in Japan. Second, one hospital was unable to provide an objective assessment of urinary catheter appropriateness and was thus excluded from appropriateness calculations. Third, we did not assess inter-observer variability. However, observers were all provided the same training and were encouraged to communicate to address any issues they ran into during the study period. Fourth, we did not collect any patient-level characteristics other than factors directly related to their urinary catheter, such as location of catheter placement. Fifth, urinary catheter presence and indication was only assessed for 3 work weeks (Monday–Friday) over 3 months on each unit. Therefore, our data may not reflect urinary catheter usage on weekends or during other times of the year.

Despite its limitations, this multicenter study provides epidemiological information about the appropriate use of urinary catheters in Japanese non-ICU wards. While use of urinary catheters was comparable to other studies, a high proportion of urinary catheter days were deemed inappropriate. Use of a multimodal intervention to promote prompt removal of catheters as soon as they are no longer appropriate may be needed to improve appropriate catheter use and reduce CAUTI risk in this setting.

## Supplementary Information

**Additional file 1: Appendix S1.** Data collection tool.

## Data Availability

The datasets generated and analysed during the current study are available from the corresponding author on reasonable request.
