# Supplementary material for: Prevalence and appropriateness of indwelling urinary catheters in Japanese hospital wards: a multicenter point prevalence study
Source: BMC Infect Dis. 2022 Feb 21;22:175. doi: 10.1186/s12879-022-07162-3 (PMC8862324; doi:10.1186/s12879-022-07162-3)
Supplement: Supplementary file 1 — Additional file 1: Appendix S1. Data collection tool. [file 12879_2022_7162_MOESM1_ESM.pdf]

Hosptial: _______________ Unit:____________

Urinary Catheter Prevalence

Total No. of pts on unit:____________ No. of Caths: _______

Date:_____________ Start Time:________ End Time: ________ Observer: __________

	Rm/Bed No.	Last Name	Medical Record Number	RN's rationale for Catheter TODAY1	Comments	*Medical Record Chart Review Items* Was Catheter Documented in Medical Record (MR) Today?		Reviewer's Indication, 1-18	Written Order?		Inserted Where?	Inserted When?
1						Yes	No		Yes	No		
2						Yes	No		Yes	No		
3						Yes	No		Yes	No		
4						Yes	No		Yes	No		
5						Yes	No		Yes	No		
6						Yes	No		Yes	No		
7						Yes	No		Yes	No		
8						Yes	No		Yes	No		

[1]This is the rationale from the bedside nurse's point of view. Enter number 1 - 18 from below.

APPROPRIATE URINARY CATHETER INDICATIONS

1. Acute urinary retention or bladder outlet obstruction
2. Need accurate I's and O's in critically ill patient
3. Perioperative use (**Unless patient meets criteria 4, 5, 6, or 7, catheter to be removed post-before transport to unit)**
   4. Urologic Surgery or surgery on contiguous structures of genitourinary tract
   5. Anticipated prolonged duration of surgery
   6. Pt anticipated to receive large-volume infusions or diuretics during surgery
   7. Need for intraoperative monitoring of urinary output
8. To assist in healing of open sacral or perineal wounds in incontinent patients*
9. Patient requires prolonged immobilization (e.g., unstable thoracic or lumbar spine, or multiple traumatic injuries such as pelvis fracture)
10. To improve comfort care for end of life care
   10a. Longterm indwelling catheter (includes suprapubic) or post-operative procedure

NON-APPROPRIATE INDICATIONS MAY INCLUDE:

11. Incontinence
12. Immobility
13. Monitoring I's and O's in non-critically ill pt
14. Patient request
15. Convenience
16. Confusion
17. No apparent reason
18. Other (describe in Comments)
